# Supplementary material for: Integrating end-to-end learning with deep geometrical potentials for ab initio RNA structure prediction
Source: Nat Commun. 2023 Sep 16;14:5745. doi: 10.1038/s41467-023-41303-9 (PMC10505173; doi:10.1038/s41467-023-41303-9)
Supplement: Supplementary file 1 — Supplementary Information [file 41467_2023_41303_MOESM1_ESM.pdf]

Supplementary Materials for

Integrating end-to-end learning with deep geometrical potentials for ab initio RNA structure prediction

Yang Li, Chengxin Zhang, Chenjie Feng, Robin Pearce, P. Lydia Freddolino, Yang Zhang

Supplementary Tables

Table S1. Comparison of RNA base pairing related metrics between DRfold and the control methods on the test set with sequence identity cutoffs of 90% and 80% to the training set. The bold fonts highlight the best performing method in each category.

Methods	DI	INF_all	INF_wc	INF_nwc	INF_stack
Sequence identity cutoff <90%					
3dRNA	40.30	0.586	0.633	0.067	0.597
FARFAR2	43.92	0.604	0.589	0.042	0.644
RNAComposer	41.17	0.616	0.638	0.142	0.628
RNA-BRiQ	39.36	0.622	0.613	0.095	0.645
SimRNA	50.52	0.528	0.384	0.012	0.616
DRfold	**26.27**	**0.708**	**0.768**	**0.155**	**0.711**
Sequence identity cutoff <80%					
3dRNA	36.68	0.581	0.625	0.046	0.593
FARFAR2	41.72	0.596	0.575	0.033	0.638
RNAComposer	46.76	0.605	0.616	0.122	0.622
RNA-BRiQ	43.41	0.616	0.599	0.069	0.641
SimRNA	40.90	0.525	0.377	0.000	0.612
DRfold	**31.63**	**0.673**	**0.722**	**0.126**	**0.678**

Table S2. Comparison of RNA local torsion angle parameters between DRfold and the control methods. The bold fonts highlight the best performing method in each category.

**Methods**	**MCQ**	**Handedness score**
3dRNA	0.598	0.636
FARFAR2	0.491	0.616
RNAComposer	0.533	0.611
RNA-BRiQ	**0.449**	0.648
SimRNA	0.531	0.563
DRfold	0.553	**0.730**

Table S3. Benchmark results of the 6 end-to-end models and their ensemble. The *P*-values were computed using two-tailed Student's t-tests. The bold fonts highlight the best performing method in each category.

Models	Mean TM-score	Median TM-score	*P*-value
Model 1	0.395	0.335	2.4e-02
Model 2	0.398	0.331	6.6e-02
Model 3	0.393	0.320	1.4e-02
Model 4	0.394	0.346	4.5e-02
Model 5	0.405	0.332	1.8e-01
Model 6	0.397	0.342	4.0e-02
Ensemble	**0.417**	**0.372**	-

Table S4. Performance comparison of DRfold without secondary structure feature, and with secondary structures predicted by default (consensus of RNAfold and PETfold), SPOT-RNA, and Ground-Truth secondary structure. MCC refers to the Matthews correlation coefficient between the predicted and target secondary structure assignments.

**SS prediction methods**	**MCC**	**TM-score**	**RMSD (Å)**
Without SS	-	0.295	21.10
Default	0.678	0.439	14.49
SPOT-RNA	0.727	0.433	13.61
Ground-Truth	1.000	0.443	13.17

Table S5. Performance comparison of single end-to-end component of the DRfold pipeline with secondary structures predicted by default (consensus of RNAfold and PETfold), SPOT-RNA, and Ground-Truth secondary structure. For SPOT-RNA and Ground-Truth feature, we also report the results based on the models retrained by the corresponding features.

**Testing SS feature**	**TM-score**	**RMSD (Å)**
Default	0.405	13.89
SPOT-RNA	0.405	14.23
SPOT-RNA (Retrained)	0.404	13.67
Ground-Truth	0.423	13.02
Ground-Truth (Retrained)	0.426	12.70

Table S6. Comparison of RNA structure validity parameters at different steps of structural refinement. Clash score was calculated as the number of serious clashes per 1000 atoms, obtained from the MolProbity program. RMS (bond) and RMS (angles) are the root mean square deviations of bond lengths and torsion angles of the DRfold models from their restrained ideal values. Refinement Step 1 refers to the application of Arena to construct full-atom models. Refinement Step2 refers to the application of OpenMM MD simulation package to refine the full-atom models. “Experimental” refers to the target structures in the PDB.

	Clash score	RMS (bond)	RMS (angles)	MolProbity score
Raw DRfold	224.15	0.06	10.77	3.95
Refinement Step 1	82.79	0.05	6.59	3.41
Refinement Step 2	18.57	0.03	4.21	2.83
Experimental	7.25	0.01	1.13	2.42

Table S7. Overall performance of different RNA structure prediction methods on 40 test RNAs. Methods were split into two categories depending on whether they were trained on single sequences or multiple sequence alignments (MSA), while the 'Hybrid' at the bottom row refers to the hybrid approach using the geometric restraints of DeepFoldRNA to guide the DRfold folding simulations. P-values are two-tailed Student's t-test calculated between DRfold and each individual control methods. The bold fonts highlight the best performing method in each category.

Starting from	Methods	TM-score (p-value)	RMSD (p-value)
Single sequence	3DRNA	0.251 (5.79E-07)	20.53 (7.35E-05)
	SimRNA	0.196 (2.64E-08)	23.88 (6.14E-07)
	BRiQ	0.216 (2.47E-07)	22.88 (3.34E-07)
	FARFAR2	0.203 (4.35E-08)	22.48 (3.72E-07)
	RNAcomposer	0.239 (1.05E-06)	20.80 (1.90E-04)
	FARFAR2+ARES	0.195 (2.53E-08)	22.82 (1.35E-06)
	DRfold	**0.435**	**14.44**
MSA	DeepFoldRNA	**0.485** (1.66E-02)	12.19 (1.90E-01)
	RhoFold	0.420 (4.40E-01)	11.57 (2.34E-02)
	RoseTTAFoldRNA	0.428 (5.89E-01)	14.61 (8.36E-01)
	trRosettaRNA	0.474 (9.75E-02)	**10.94** (8.80E-02)
Hybrid	DRfold/DeepFoldRNA Potential	**0.501** (1.66E-05)	**10.65** (4.41E-05)

Table S8. Z-score based relative group performance of first models for RMSD with penalty thresholds of -2.0 and 0.0, respectively.

SUM Z-score > -2.0			SUM Z-score > -0.0		
Rank	Group ID	SUM Zscore	Rank	Group ID	SUM Zscore
1	Chen	13.46	1	Chen	15.00
2	AIchemy_RNA2	13.40	2	AIchemy_RNA2	14.48
3	RNApolis	10.74	3	RNApolis	11.22
4	Yang-Server	06.01	4	GeneSilico	08.14
**5**	**rDP**	**05.72**	5	Yang-Server	06.68
6	CoMMiT-server	03.63	**6**	**rDP**	**06.18**
7	CoMMiT-human	03.48	7	AIchemy_RNA	05.73
8	UltraFold	02.14	8	UltraFold	05.73
9	Yang	01.97	9	Yang- Multimer	05.27
10	Kiharalab	01.50	10	CoMMiT-server	05.21
11	UltraFold_Server	00.68	11	CoMMiT-human	05.11
12	GeneSilico	00.56	12	Yang	04.83
13	AIchemy_RNA	00.38	13	Kiharalab	04.69
14	Yang- Multimer	-00.39	14	UltraFold_Server	04.31
15	Coqualia	-02.41	15	SoutheRNA	03.42
16	SoutheRNA	-02.68	16	LCBio	03.29
17	LCBio	-02.90	17	Coqualia	03.24
18	BAKER	-04.08	18	DF_RNA	02.67
19	Rookie	-04.49	19	BAKER	02.64
20	Manifold-E	-06.69	20	nucE2E	02.62
21	SHT	-06.78	21	Rookie	01.91
22	GinobiFold	-06.97	22	CoDock	01.75
23	FoldEver	-07.49	23	AIchemy_LIG	01.74
24	GWxraylab	-07.91	23	AIchemy_LIG3	01.74
25	FoldEver-Hybrid	-08.61	23	AIchemy_LIG2	01.74
26	Manifold	-09.04	26	PerezLab_Gators	01.53
27	DF_RNA	-10.58	27	Manifold	01.37
28	nucE2E	-11.38	28	SHT	01.23
29	CoDock	-12.25	29	FoldEver	01.20
30	Schug_Lab	-12.90	29	FoldEver-Hybrid	01.20
31	PerezLab_Gators	-16.38	31	GinobiFold	01.11
32	WL_team	-19.33	32	Venclovas	01.00
33	Graphen_Medical	-19.37	33	WL_team	00.86
34	Kiharalab_Server	-19.48	34	Manifold-E	00.66
35	Venclovas	-19.48	35	Schug_Lab	00.55
36	AIchemy_LIG	-20.26	36	Kiharalab_Server	00.49
36	AIchemy_LIG3	-20.26	37	GWxraylab	00.49
36	AIchemy_LIG2	-20.26	38	Manifold-LC-E	00.33
39	Manifold-LC-E	-21.67	39	UNRES	00.00
40	Manifold-LC	-22.53	39	Manifold-LC	00.00
41	UNRES	-24.00	39	Graphen_Medical	00.00

Table S9. Z-score based relative group performance of first models for TM-score with penalty thresholds of -2.0 and 0.0, respectively.

SUM Z-score > -2.0			SUM Z-score > -0.0		
Rank	Group ID	SUM Zscore	Rank	Group ID	SUM Zscore
1	AIchemy_RNA2	20.72	1	AIchemy_RNA2	21.35
2	Chen	16.34	2	Chen	16.42
3	RNApolis	12.44	3	RNApolis	12.91
4	GeneSilico	04.28	4	GeneSilico	10.48
5	Yang-Server	02.83	5	AIchemy_RNA	05.96
**6**	**rDP**	**02.22**	6	CoMMiT-human	04.50
7	CoMMiT-human	01.36	7	Yang-Server	04.26
8	AIchemy_RNA	01.03	8	CoMMiT-server	04.08
9	UltraFold	01.01	**9**	**rDP**	**04.03**
10	CoMMiT-server	00.94	10	UltraFold	03.63
11	Kiharalab	00.80	11	GWxraylab	03.54
12	Yang	-00.17	12	SoutheRNA	03.14
13	SoutheRNA	-00.37	13	Kiharalab	03.01
14	SHT	-00.45	14	Yang	02.96
15	GWxraylab	-00.66	15	DF_RNA	02.88
16	UltraFold_Server	-00.91	16	LCBio	02.54
17	Coqualia	-01.41	17	Coqualia	02.35
18	GinobiFold	-01.64	18	Rookie	02.35
19	Rookie	-02.58	19	Manifold	02.30
20	Manifold-E	-03.61	20	SHT	02.23
21	Yang- Multimer	-03.75	21	AIchemy_LIG	02.19
22	Manifold	-03.86	21	AIchemy_LIG3	02.19
23	LCBio	-04.42	21	AIchemy_LIG2	02.19
24	BAKER	-04.56	24	Yang- Multimer	02.17
25	DF_RNA	-07.60	25	Venclovas	02.11
26	FoldEver	-12.15	26	GinobiFold	01.81
27	Schug_Lab	-12.49	27	UltraFold_Server	01.79
28	FoldEver-Hybrid	-13.39	28	BAKER	01.63
29	Kiharalab_Server	-13.87	29	Manifold-E	01.56
30	CoDock	-14.93	30	PerezLab_Gators	01.41
31	Graphen_Medical	-15.42	31	CoDock	01.14
32	PerezLab_Gators	-16.16	32	Kiharalab_Server	01.08
33	nucE2E	-16.17	33	WL_team	00.73
34	Venclovas	-17.89	34	Schug_Lab	00.48
35	AIchemy_LIG	-19.81	35	nucE2E	00.39
35	AIchemy_LIG3	-19.81	36	Manifold-LC-E	00.00
35	AIchemy_LIG2	-19.81	36	UNRES	00.00
38	WL_team	-21.27	36	Manifold-LC	00.00
39	Manifold-LC	-22.16	36	FoldEver	00.00
40	Manifold-LC-E	-22.19	36	FoldEver-Hybrid	00.00
41	UNRES	-22.68	36	Graphen_Medical	00.00

Table S10. Group performance of first models for average RMSD and TM-score, respectively. Groups that submitted models for all targets were considered.

Rank	Group ID	RMSD (Å)	Rank	Group ID	TM-score
1	AIchemy_RNA2	14.03	1	AIchemy_RNA2	0.485
2	Chen	15.48	2	Chen	0.432
3	RNApolis	15.90	3	RNApolis	0.401
**4**	**rDP**	**21.60**	4	Yang-Server	0.305
5	Yang-Server	21.85	5	UltraFold	0.295
6	UltraFold	23.12	6	CoMMiT-human	0.294
7	UltraFold_Server	23.43	7	CoMMiT-server	0.291
8	CoMMiT-server	23.55	8	Kiharalab	0.291
A	CoMMiT-human	23.72	**9**	**rDP**	**0.288**
10	Kiharalab	24.46	10	UltraFold_Server	0.286
11	Coqualia	25.75	11	SoutheRNA	0.281
12	SoutheRNA	28.15	12	SHT	0.280
13	SHT	28.95	13	GWxraylab	0.276
14	GinobiFold	29.65	14	Coqualia	0.273
15	FoldEver	31.20	15	GinobiFold	0.270
16	GWxraylab	31.61	16	Manifold	0.246
17	Manifold-E	31.97	17	Manifold-E	0.242
18	Manifold	32.98	18	FoldEver	0.196
19	Graphen_Medical	41.80	19	Graphen_Medical	0.171
20	Kiharalab_Server	82.57	20	Kiharalab_Server	0.164

Supplementary Figures

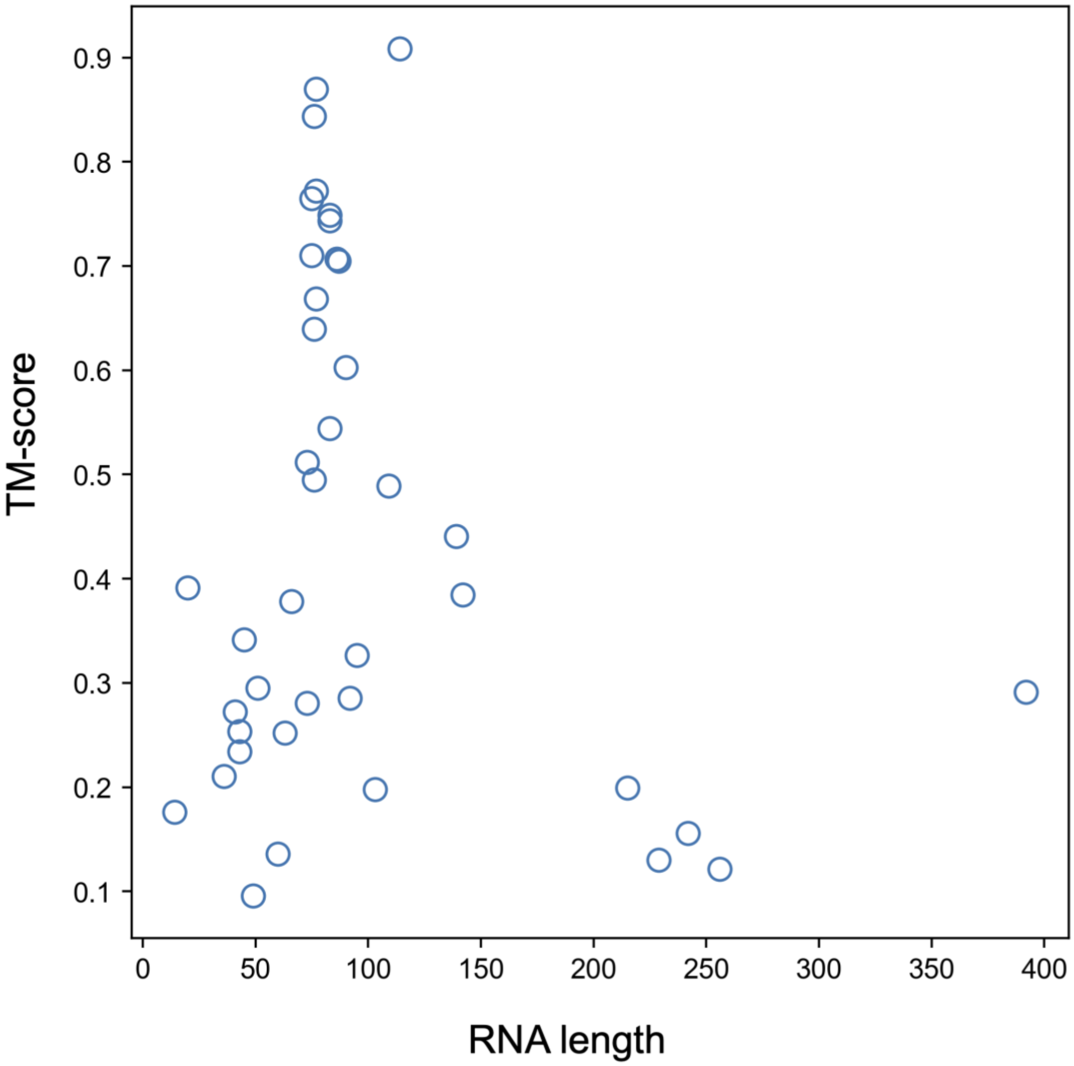

Figure S1. TM-score of predicted structures versus the RNA length

7o80AT

```
Native:  ((((.((..(((.....[....))).(((((.......)))))..(((....)))...(((((..]....))))))).))))....
DRfold:  ((((.((..(((...&.[....)))((((((.......))))))((((....))))..(((((..]....))))))).))))....
```

7o7zAH

```
Native:  .........................(((((((((...[[[[[..)))))))))((((((((((((....)).))).)))))))....]]]]]...
DRfold:  .........................(((((((((....[.[...)))))))))(.((((((((.........))).))))).)......]]....
```

Figure S2. Comparison of the secondary structures for the native and DRfold structures for targets 7o80AT and 7o7zAH, respectively. Red color highlights the assigned pseudoknots.

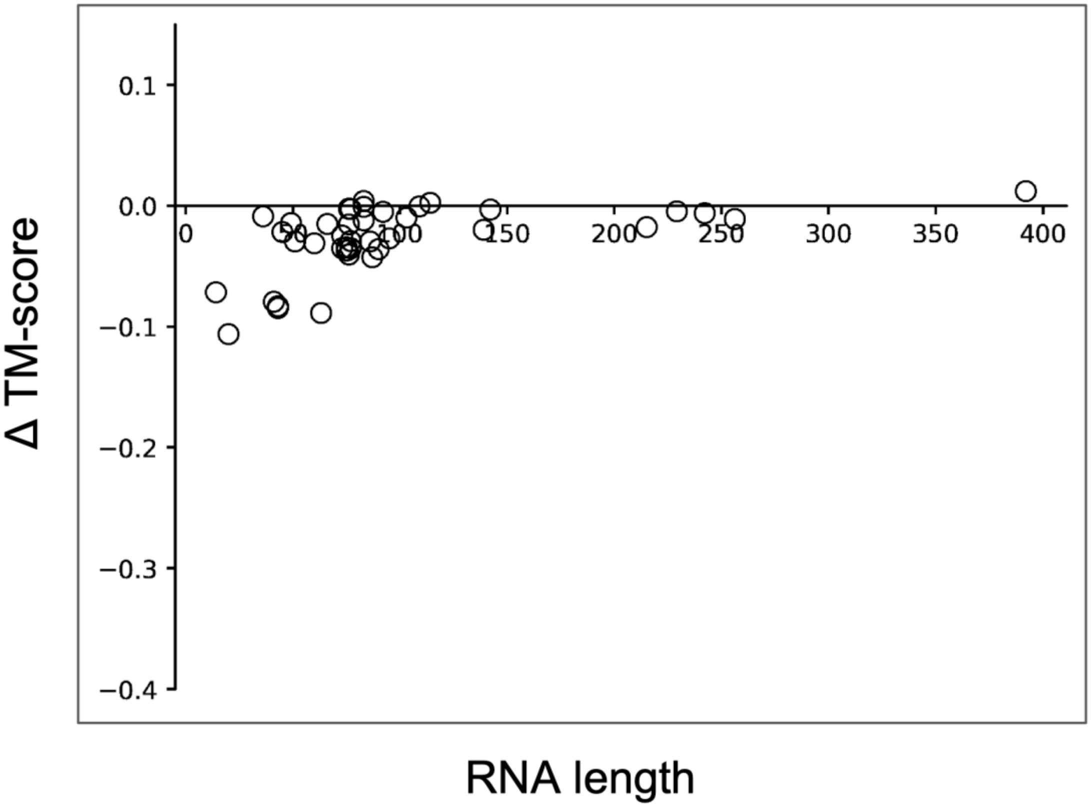

Figure S3. The difference of TM-score versus the RNA length without the geometry potentials compared to the full pipeline (negative values indicate worse performance for the reduced pipeline).

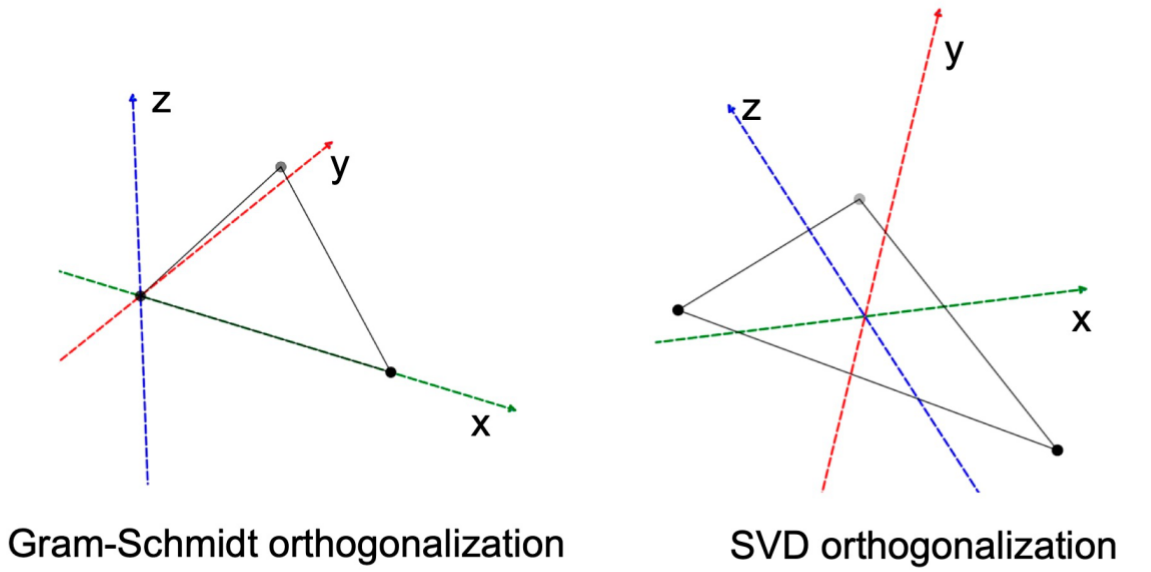

Figure S4. An illustration of the difference between Gram-Schmidt and SVD orthogonalization.

Supplementary Texts

Text S1. A brief introduction of the configurations of the control methods

Multiple RNA structure prediction methods have been used as control methods in our benchmark tests. Among the traditional approaches, RNAComposer[1], FARFAR2[5] and 3dRNA[2] are the representative fragment assembly methods, while RNA-BRiQ[3] and SimRNA[4] are the two representative *ab initio* RNA structure prediction methods.

The predictions of RNAComposer and 3dRNA were directly obtained by feeding their web servers with query sequences and secondary structure predictions from RNAfold[6]. All other options were kept unchanged. More specifically, for 3dRNA, the "_routine" and "_ss_method" parameters were "assemble" and "RNAfold" respectively.

RNA-BRiQ, SimRNA, and FARFAR2 were installed locally and provided with sequence information and predicted secondary structures from RNAfold. The "BRiQ_Predict" command was used to predict RNA structures for RNA-BRiQ. For SimRNA, the "SimRNA" command was first used with the "-E" option set to 10. The "clustering" command was then used for clustering, followed by the "SimRNA_trafl2pdbs" command with the "AA" option to extract final predictions. For FARFAR2, the "rna_denovo" command was used with default settings and a maximum running time of 72 hours. Final predictions were selected based on the minimal energy.

Additionally, 5 deep learning based methods, including ARES[7], DeepFoldRNA[8], RhoFold[9], RoseTTAFoldRNA[10] and trRosettaRNA[11], were also considered for benchmark. All these methods were installed locally with the default settings. Note that ARES was configured to perform the conformation selection from the structures generated by FARFAR2.

Supplementary References

1 Biesiada, M., Pachulska-Wieczorek, K., Adamiak, R. W. & Purzycka, K. J. RNAComposer and RNA 3D structure prediction for nanotechnology. *Methods* **103**, 120-127, doi:https://doi.org/10.1016/j.ymeth.2016.03.010 (2016).

2 Zhao, Y. *et al.* Automated and fast building of three-dimensional RNA structures. *Scientific Reports* **2**, 734, doi:10.1038/srep00734 (2012).

3 Xiong, P., Wu, R., Zhan, J. & Zhou, Y. Pairing a high-resolution statistical potential with a nucleobase-centric sampling algorithm for improving RNA model refinement. *Nature Communications* **12**, 2777, doi:10.1038/s41467-021-23100-4 (2021).

4 Boniecki, M. J. *et al.* SimRNA: a coarse-grained method for RNA folding simulations and 3D structure prediction. *Nucleic acids research* **44**, e63, doi:10.1093/nar/gkv1479 (2016).

5 Watkins, A. M., Rangan, R. & Das, R. FARFAR2: Improved De Novo Rosetta Prediction of Complex Global RNA Folds. *Structure* **28**, 963-976 e966, doi:10.1016/j.str.2020.05.011 (2020).

6 Lorenz, R. *et al.* ViennaRNA Package 2.0. *Algorithms for Molecular Biology* **6**, 26, doi:10.1186/1748-7188-6-26 (2011).

7 Townshend Raphael, J. L. *et al.* Geometric deep learning of RNA structure. *Science* **373**, 1047-1051, doi:10.1126/science.abe5650 (2021).

8 Pearce, R., Omenn, G. S. & Zhang, Y. De Novo RNA Tertiary Structure Prediction at Atomic Resolution Using Geometric Potentials from Deep Learning. *bioRxiv*, 2022.2005. 2015.491755 (2022).

9 Shen, T. *et al.* E2Efold-3D: End-to-End Deep Learning Method for accurate de novo RNA 3D Structure Prediction. *arXiv preprint arXiv:2207.01586* (2022).

10 Baek, M., McHugh, R., Anishchenko, I., Baker, D. & DiMaio, F. Accurate prediction of nucleic acid and protein-nucleic acid complexes using RoseTTAFoldNA. *bioRxiv*, 2022.2009. 2009.507333 (2022).
11 Feng, C. *et al.* Accurate de novo prediction of RNA 3D structure with transformer network. *bioRxiv*, 2022.2010. 2024.513506 (2022).
