# Supplementary material for: Integrating end-to-end learning with deep geometrical potentials for ab initio RNA structure prediction
Source: Nat Commun. 2023 Sep 16;14:5745. doi: 10.1038/s41467-023-41303-9 (PMC10505173; doi:10.1038/s41467-023-41303-9)
Supplement: Supplementary file 3 — Reporting Summary [file 41467_2023_41303_MOESM3_ESM.pdf]

nature portfolio

Corresponding author(s): Yang Zhang

Last updated by author(s): Aug 1, 2023

Reporting Summary

Nature Portfolio wishes to improve the reproducibility of the work that we publish. This form provides structure for consistency and transparency in reporting. For further information on Nature Portfolio policies, see our Editorial Policies and the Editorial Policy Checklist.

Statistics

For all statistical analyses, confirm that the following items are present in the figure legend, table legend, main text, or Methods section.

n/a	Confirmed	
☐	☒	The exact sample size (*n*) for each experimental group/condition, given as a discrete number and unit of measurement
☐	☒	A statement on whether measurements were taken from distinct samples or whether the same sample was measured repeatedly
☐	☒	The statistical test(s) used AND whether they are one- or two-sided<br>*Only common tests should be described solely by name; describe more complex techniques in the Methods section.*
☐	☒	A description of all covariates tested
☒	☐	A description of any assumptions or corrections, such as tests of normality and adjustment for multiple comparisons
☐	☒	A full description of the statistical parameters including central tendency (e.g. means) or other basic estimates (e.g. regression coefficient) AND variation (e.g. standard deviation) or associated estimates of uncertainty (e.g. confidence intervals)
☐	☒	For null hypothesis testing, the test statistic (e.g. *F*, *t*, *r*) with confidence intervals, effect sizes, degrees of freedom and *P* value noted<br>*Give P values as exact values whenever suitable.*
☒	☐	For Bayesian analysis, information on the choice of priors and Markov chain Monte Carlo settings
☒	☐	For hierarchical and complex designs, identification of the appropriate level for tests and full reporting of outcomes
☒	☐	Estimates of effect sizes (e.g. Cohen's *d*, Pearson's *r*), indicating how they were calculated

Our web collection on statistics for biologists contains articles on many of the points above.

Software and code

Policy information about availability of computer code

Data collection	cd-hit (4.8.1) was used for sequence clustering. US-align (1.0) was used for TM-score calculation. RNA_assessment (https://github.com/RNA-Puzzles/RNA_assessment) was used for INF and DI calculation.
Data analysis	Data were analyzed using Numpy v.1.20.3 (https://github.com/numpy/numpy), SciPy v.1.7.1 (https://www.scipy.org/), and Matplotlib v.3.4.3 (https://github.com/matplotlib/matplotlib). Structures were visualized by Pymol v.2.3.0 (https://github.com/schrodinger/pymol-open-source) and UCSF ChimeraX v.1.5 (https://www.cgl.ucsf.edu/chimerax/).

For manuscripts utilizing custom algorithms or software that are central to the research but not yet described in published literature, software must be made available to editors and reviewers. We strongly encourage code deposition in a community repository (e.g. GitHub). See the Nature Portfolio guidelines for submitting code & software for further information.

Data

Policy information about availability of data

All manuscripts must include a data availability statement. This statement should provide the following information, where applicable:

- Accession codes, unique identifiers, or web links for publicly available datasets
- A description of any restrictions on data availability
- For clinical datasets or third party data, please ensure that the statement adheres to our policy

All input data are freely available from public sources. We show structures of 7OX9 and 7MRL obtained by through four-digit accession codes in the Protein Data Bank repository (https://www.rcsb.org/). RNA structures for training were collected from PDB (https://www.wwpdb.org/ftp/pdb-ftp-sites).

Research involving human participants, their data, or biological material

Policy information about studies with human participants or human data. See also policy information about sex, gender (identity/presentation), and sexual orientation and race, ethnicity and racism.

Reporting on sex and gender	N/A
Reporting on race, ethnicity, or other socially relevant groupings	N/A
Population characteristics	N/A
Recruitment	N/A
Ethics oversight	N/A

Note that full information on the approval of the study protocol must also be provided in the manuscript.

Field-specific reporting

Please select the one below that is the best fit for your research. If you are not sure, read the appropriate sections before making your selection.

☒ Life sciences ☐ Behavioural & social sciences ☐ Ecological, evolutionary & environmental sciences

For a reference copy of the document with all sections, see nature.com/documents/nr-reporting-summary-flat.pdf

Life sciences study design

All studies must disclose on these points even when the disclosure is negative.

Sample size	Two test sets were used in this study. The first set contains 40 non-redundant RNA structures with lengths from 14 to 392 nucleotides, which were collected from sequence cluster centers (sequence identity cutoff of 90%) of solved structures deposited in or after the year 2021 in the PDB. The second test set contains all 12 RNA targets in CASP15.
Data exclusions	No data were excluded.
Replication	All source code and benchmark sets are available, the results are reproducible.
Randomization	All the set sequences, as well as their cluster members were excluded from the training set. The clustering was performed by software cd-hit (4.8.1),
Blinding	The investigators were not blind to the first test set, but were blind to the 12 RNA targets in CASP15. The proposed method was tested in CASP15 where all participants are blind to the structures of the test targets.

Reporting for specific materials, systems and methods

We require information from authors about some types of materials, experimental systems and methods used in many studies. Here, indicate whether each material, system or method listed is relevant to your study. If you are not sure if a list item applies to your research, read the appropriate section before selecting a response.

Materials & experimental systems

n/a	Involved in the study
☒	☐ Antibodies
☒	☐ Eukaryotic cell lines
☒	☐ Palaeontology and archaeology
☒	☐ Animals and other organisms
☒	☐ Clinical data
☒	☐ Dual use research of concern
☒	☐ Plants

Methods

n/a	Involved in the study
☒	☐ ChIP-seq
☒	☐ Flow cytometry
☒	☐ MRI-based neuroimaging
